# Feeling labeled, judged, lectured, and rejected by family and friends over depression: Cautionary results for primary care clinicians from a multi-centered, qualitative study

**DOI:** 10.1186/1471-2296-13-64

**Published:** 2012-06-29

**Authors:** Erik Fernandez y Garcia, Paul Duberstein, Debora A Paterniti, Camille S Cipri, Richard L Kravitz, Ronald M Epstein

**Affiliations:** 1 Department of Pediatrics, University of California, Davis, School of Medicine, Sacramento, CA, USA; 2 Center for Communication and Disparities Research and the Rochester Health Decision Making Group, University of Rochester Medical Center, Rochester, New York, USA; 3 Department of Psychiatry, University of Rochester Medical Center, Rochester, NY, USA; 4 Center for Healthcare Policy and Research, University of California, Davis, School of Medicine, Sacramento, CA, USA; 5 Department of Internal Medicine, University of California, Davis, School of Medicine, Sacramento, CA, USA; 6 Department of Sociology, University of California, Davis, Sacramento, CA, USA; 7 Departments of Family Medicine and Oncology, University of Rochester School of Medicine and Dentistry, Rochester, NY, USA

**Keywords:** depression, disclosure, norms, patient-provider communication, social support, qualitative analysis

## Abstract

**Background:**

Family and friends may help patients seek out and engage in depression care. However, patients’ social networks can also undermine depression treatment and recovery. In an effort to improve depression care in primary care settings, we sought to identify, categorize, and alert primary care clinicians to depression-related messages that patients hear from friends and family that patients perceive as unhelpful or detrimental.

**Methods:**

We conducted 15 focus groups in 3 cities. Participants (n = 116) with a personal history or knowledge of depression responded to open-ended questions about depression, including self-perceived barriers to care-seeking. Focus group conversations were audio-recorded and analyzed using iterative qualitative analysis.

**Results:**

Four themes emerged related to negatively-received depression messages delivered by family and friends. Specifically, participants perceived these messages as making them feel labeled, judged, lectured to, and rejected by family and friends when discussing depression. Some participants also expressed their interpretation of their families’ motivations for delivering the messages and described how hearing these messages affected depression care.

**Conclusions:**

The richness of our results reflects the complexity of communication within depression sufferers’ social networks around this stigmatized issue. To leverage patients’ social support networks effectively in depression care, primary care clinicians should be aware of both the potentially beneficial and detrimental aspects of social support. Specifically, clinicians should consider using open-ended queries into patients’ experiences with discussing depression with family and friends as an initial step in the process. An open-ended approach may avoid future emotional trauma or stigmatization and assist patients in overcoming self-imposed barriers to depression discussion, symptom disclosure, treatment adherence and follow-up care.

## Background

Primary care is the *de facto* setting for identifying and treating most cases of adult depression [1,2]. Given the stigma attached to mental disorders [3], societal constraints on personal disclosure [4], and public concern about treatment effectiveness and toxicity [5], primary care practitioners (PCPs) have been placed in the unenviable position of being tasked with identifying and treating depression in the face of patient reticence. We are currently examining the effectiveness of health communications interventions designed to improve depression care by enhancing disclosure of depression in the primary care setting. Before developing the interventions, focus group interviews were conducted with individuals having first-hand knowledge of depression. Prior reports from these focus groups have focused on difficulties surrounding disclosure of depression relating to patient characteristics [6], barriers in physician-patient interaction and organizational systems [7], and aspects of the subjective experience of depression that hinder depressed individuals’ ability to recognize what is wrong, attach a name to the experience, and find a meaningful explanation for it [8].

In the course of analyzing our focus group data, we were struck by our participants’ observations of friends and relatives offering comments, often well-intentioned, that were negatively received. In the context of bereavement, messages of “support”, such as “*Life is for living,” “It is time you got over it,”* or “*Time heals all wounds,”* have had similar deleterious effects [9]. Perhaps because depressed patients rarely speak openly or spontaneously about their hurtful exchanges with friends and relatives [10], these types of comments, as related to depression, have not yet been systematically exposed.

Documenting these messages has important implications for family-centered PCPs’ approach to depression identification and treatment. Depression imposes challenges and burdens not just on depressed individuals but also on members of their social network [11,12]. Many families rise to these challenges and help patients seek and follow through with care. However, the burgeoning literature on “negative social support” provides a counter-balance to literature on the positive aspects of the involvement of family and friends; it suggests that not only can communication from relatives and friends be experienced as hurtful, undermining, dismissing or damaging [13-15], but can also result in patients experiencing even lower levels of social support [16-18]. The relationships between patients and primary care clinicians (PCPs, nurses, and/or care managers) may assume a heightened level of importance for depressed patients in primary care who experience such negative social support. If unaware of negative messages from friends and family, clinicians may unwittingly reinforce such messages. If clinicians are aware of the potential negative effects of such messages, they can consciously work collaboratively [19,20] to de-toxify these messages and thus, potentially, improve the likelihood that patients will receive appropriate care [21,22].

Our primary goal in this analysis was to categorize the negatively received depression-related messages that depressed patients might hear from friends and family in contrast to the generally accepted supportive messages that these social networks provide. Further, we sought to understand the potential impact of these messages on patients; to suggest ways in which clinicians can understand the complex nature of patients’ social support networks around depression; and suggest ways clinicians can start to build therapeutic relationships with depressed patients that may mitigate the negative effects of the social messages that patients are hearing.

## Methods

### Study design

The data obtained for the present study were gathered as part of the formative research of a larger project. The focus of this larger project is to develop and evaluate office-based interventions to encourage patients to disclose depressive symptoms, allowing the patient and the clinical team to make informed collaborative decisions about appropriate treatment [21,22]. As part of the formative research, we convened 15 focus group interviews of people who reported experience with depression in themselves and/or close relatives. We chose focus group methods to use interactions among participants that would capture the diversity of patient care-seeking experiences in a supportive environment [23]. The team developed guiding questions about individual, interpersonal, and organizational barriers to care-seeking that were informed by theories of health behavior change and illness cognition [24]. These study questions have been published elsewhere [8].

The Institutional Review Boards at the three study sites (Rochester, New York; Austin, Texas; and Sacramento, California) approved all study procedures. Participants’ eligibility was assessed after they responded to a variety of recruitment strategies, including the internet message board “Craigslist.com,” flyers, neighborhood canvassing, and working with community leaders and clergy. Recruitment materials included only neutral descriptions of the study, such as it being “about why people might or might not seek treatment for depression” and “about your health care experiences with depression.” Eligibility criteria stated that potential study participants were to be English-speaking men and women, ages 25–64 years, who reported a history of depression in self or “in a close friend or relative.” While participants were purposively sampled to achieve maximum variation and representativeness by gender, age, and racial/ethnic background, we focused on working-aged adults because this group is both understudied and likely to contribute disproportionately to the economic burden of depression [25]. Focus groups were stratified by gender because prior research has identified gender differences in care-seeking experiences [26]. Focus groups were also stratified by median household income level corresponding to participant zip code, as a proxy for socioeconomic status, and was designated as “low” or “middle” based on their percentiles relative to the median. Written informed consent was obtained from all participants. Research assistants trained in the protection of human subjects, reviewed the study procedures by telephone with respondents to recruiting efforts. Potential participants were sent a hardcopy of the study procedure and consent form by mail. Once at the focus group, group moderators reviewed the study procedures and the consent form and allowed time for questions about consent and participation. Once all questions were addressed, participants gave consent by signing the informed consent form. The focus group began once all consent forms were signed and collected. After conducting 3 pilot focus groups, we held 12 more groups (4 at each study site) between February and April 2008. Study participants received a $35 stipend for participation in group discussions that lasted 75–110 min. Focus group discussions were digitally recorded and then transcribed verbatim by a professional transcriptionist in manner ensuring anonymity of participants in the resulting transcript.

### Data analysis

After verifying the accuracy of each focus group transcript, general themes relating to the focus group guiding questions were identified in each of the 15 transcripts by two of the authors (DAP and CSC). The study team then used these to identify conceptual categories important to understanding individuals’ experiences of depression and care-seeking through a series iterative review of transcripts, team meetings, and discussion. Conceptual categories were then organized into larger themes by group consensus. Two of the authors (DAP and CSC) systematically reviewed each transcript and coded thematically relevant segments using the qualitative software EthnoNotes Version 1.0. The software was later used to facilitate searches for thematically illustrative quotations and their contexts.

In the second phase of analysis, a data analysis team focused on stigma and negative messaging (EFG and PD) iteratively reviewed and discussed codes pertaining to “cognitive and communicative processes that hindered or enabled discussion of depression-related symptoms,” one of the themes by the team during the iterative thematic analysis. The second phase analysis team members reviewed the transcripts and thematic categories and then developed sub-codes specific to “negative cognitive and communicative processes related to social support.” The team then examined each of the 15 transcripts for the codes and sub-codes, generating hypotheses about connections between and across codes and sub-codes. The team extracted representative quotes and identified broad themes based on linkages hypothesized between codes and sub-codes. Disconfirming data were used to modify themes and refine hypotheses about the links between themes. Co-investigators (RME and RLK) not involved in previous phases of the analysis audited the results for consistency, clarity, and comprehensiveness.

## Results

These results are reported in adherence to the R.A.T.S. guidelines for qualitative research [27]. One hundred eighty-three eligible people responded to our recruitment efforts; 37 were unavailable or ineligible due to age or income. We were able to accommodate 116 (64 %) into one of 15 scheduled focus groups (Table 1).

**Table 1:** Demographic characteristics of participants

**Variable**	**Participants (n = 116)**	
	**No.**	**%**
Respondent type		
Personal Experience	56	48
Family member or friend	14	12
Both personal and family/friend	46	40
Sex		
Male	51	44
Female	65	56
Age, years		
25-45	54	47
46-64	61	53
Not reported	1	1
Race/Ethnicity		
White	70	60
African-American/Black	19	16
Hispanic	16	14
Asian	3	3
Other	8	7
Educational Attainment		
Some high school	5	4
High school graduate/GED	18	15
Technical school/some college	38	33
College graduate	38	33
Postgraduate	16	14
Not reported	1	1
Annual household income (US Dollars)		
>80,000	13	11
50,001-80,000	15	13
40,001-50,000	13	11
30,001-40,000	10	9
20,001-30,000	14	12
<=20,000	50	43
Not reported	1	1

*Some percentages do not add up to 100% due to rounding.

In our second phase of analysis, we identified four broad themes of codes and sub-codes relating to “negative cognitive and communicative processes related to social support”: *feeling labeled, feeling judged*, *feeling lectured* and *feeling rejected*. While these represent an over-simplification of the many potential complex interactions with social networks that PCPs may anticipate for their depressed patients, they represent a heuristic framework within which we organize our results. Although presented sequentially, we do not mean to imply a causal pathway between themes for any given participant or that the thought processes reflected by participants’ statements progressed hierarchically from one theme to another. Furthermore, we have chosen to present specific quotes, of the many available, as examples of a given theme and do not intend imply mutually exclusive experiences for a given participant. These results are summarized in Table 2.

**Table 2:** A summary of the main findings: themes, codes and sub-codes used to define themes, and example quotes

**Themes**	**Codes and Sub-codes Defining Themes**	**Example Quotes**
Feeling Labeled	Impact of Statements on the Participant	*As far as discussin' [depression] with family members…I wouldn't feel comfortable doing that….they might put a label on me, you know, I’m crazy or something like that.*
	1. Emotional Health	
	2. Motivation to Discuss Depression	
Feeling Judged	Family Members’ Presumed Standard for Judgment of Causes of Depressive Symptoms	*Other people are, like, “You should be so happy. You have two kids. You have a nice husband. You have this, you have that.” [My mother]’s like, “Why are you so miserable all the time?” I’m like, “I just am.”*
	1. Life Circumstances	
	2. Inheritance	
	3. Religion	
Feeling Lectured	Family Members’ Presumed Reason for Suggesting that Depressive Symptoms Can Be Voluntarily Controlled	*One thing that I really didn't want to be told, and I was told multiple times, uh, was that it's all in my head. Uh, it's all in my head, and, uh, you know, it's all in my head and get over it…it's like belittling, you know? It, it made, made me feel like I'm the one that's trying to draw attention to myself, you know, or I'm blowing it out of proportion.*
	1. Depressive Symptoms are Manufactured by the Sufferer	
	2. Depressive Symptoms are Related to Physical Symptoms	
Feeling Rejected	Type of Discussion with Family Members That Was Attempted and Rejected	*They’re tired. They’ve been tired. They’ve been living with it. They’re tired. They’re fed up. They don’t have the strength anymore…when we go to them one more time and say, “Blah, blah, blah, blah, blah.” You know, they don’t want to hear it. They, they don’t want to hear it.*
	1. Depressive Symptom Disclosure (Including Presumed Reasons for Rejection)	
	2. Depression Treatment Choices	

### Feeling labeled

Participants’ recalled feeling as if they were being labeled by family members because of their depression symptoms. Moreover, participants described how this labeling could be hurtful. For example, one participant recalled, “*My family calls me very serious. ‘You’re always so serious.’ My mom’s like ‘You’re always so serious.’ Like, no, I’m just depressed. I don’t find enjoyment in that or humor in that*.” Another remembered, “*When I had my first serious episode of depression, I got these messages from my family that, you know, ‘you’re getting lazy.’ And so I internalized a lot of shame.”*

Some participants’ statements demonstrate how this hurtful labeling may have the effect of diminishing past and future discussion of depression symptoms with family. For example, one participant recalled, “*We were never allowed to express our feelings and if, and if we did, as a male, we were called a ‘sissy.’ I remember being called ‘sissy’ all the time.”* It appears that for this participant, labeling was used as an attempt to stifle discussion of possible depression. Other participants went on to describe how fear of labeling may inhibit future discussion. For example, one participant stated, “*As far as discussin' [depression] with family members…I wouldn't feel comfortable doing that….they might put a label on me, you know, I’m crazy or something like that.”* Another participant said, “*…[I]f I go to my parents and, you know, say whether I’m sad or depressed or whatever they just classify it as you’re just depressed. You need to be on medication. Not really sitting and understanding, okay, it’s a daily struggle. I’m a single parent.”*

Other participants’ statements demonstrate how labeling could inhibit discussion of depression not only with family but also with healthcare professionals. One participant’s recollection poignantly exemplified how feeling labeled as having no symptoms produced this effect, by explaining, “*My mother wouldn’t allow anyone to take seriously that I was having problems with depression…I remember trying to say something to the pediatrician and she said, ‘No, no, no, [she] is absolutely fine. There’s nothing wrong with [name]— nothing, nothing, nothing.’… I was in counseling at school and I told them they could not tell my parents because I wasn’t allowed to have any problems.”*

### Feeling judged

Participants did not offer explanations for how or why labels were assigned to them and their depressive symptoms by members of their social networks. However, other participants did recall clear standards by which they felt judged in regard to depression. These participants’ statements reveal how they were informed by friends and relatives of their own explanatory models of depression symptoms, and how the presence or absence of these presumed causes in participants’ lives may or may not justify sufferers’ symptoms. One type of judgment reported by participants was relating depressive symptoms to life circumstances. For example, one participant remembered being told, “*But* y*ou’ve got so much to be glad for.”* Another participant stated, “*Other people are, like, ‘You should be so happy. You have two kids. You have a nice husband. You have this, you have that.’ [My mother]’s like, ‘Why are you so miserable all the time?’ I’m like, ‘I just am.’”* For this person, being judged as lacking life circumstances that would justify feeling depressed appeared to suppress further discussion and the possibility of support.

Another judgment reported by participants related to placing depressive symptoms in the context of presumed inheritance or genetic predisposition. How this understanding of the cause of depression influenced family members’ judgment of symptom legitimacy was complex and varied. For example, one participant described that their parent’s judgment appeared to stem from their own experience by recalling, “*My mother always said I’m going to be mentally ill because my mother was mentally ill.”* Another participant felt that being informed of presumed inheritance seemed to arise from a desire to ascribe blame and remembered, “*Well, you know, my mother always said that…you inherited from your dad this chemical imbalance…Well, she and my dad didn't get along. They got a divorce. But there's no reason for her to carry her resentment or try to force it down our throats when…she hasn't even mapped the human genome.”*

Participants also reported depressive symptoms being linked to religion. Some participants appeared to see this as a judgment of their culpability in depression, as one participant remembered, “*I grew up in a Catholic church and I was--felt like I was being told it was my fault. If I wasn’t, you know, if I prayed enough, if I did whatever enough, then I would be ‘okay.’”* For other participants, such blame may have inhibited their ability to discuss their symptoms. One such participant demonstrates this by responding with one word, “*Catholicism*,” to the guiding question “*What keeps you from telling people [about depression*]?”

### Feeling lectured

Participants’ statements also revealed that relatives’ and friends’ responses to their depressive symptoms could move beyond labeling and judging, to providing repeated, unilateral and unhelpful prescriptions for improving symptoms. Specifically, participants recall being told by friends and family members that feeling the depressive symptoms could consciously be controlled. Participants’ recollections obviously reflect pain at being told to stop suffering. As there might be various reasons why members of participants’ social networks felt that depressive symptoms might be under volitional control, there may similarly be various reasons why participants were often hurt by those statements. For some, it seemed that their family implied that because the symptoms were voluntarily created they could be voluntarily eliminated. One participant, for example, remembered, “*One thing that I really didn't want to be told, and I was told multiple times, uh, was that it's all in my head. Uh, it's all in my head, and, uh, you know, it's all in my head and get over it…it's like belittling, you know? It, it made, made me feel like I'm the one that's trying to draw attention to myself, you know, or I'm blowing it out of proportion.”* Another participant recalled, “*[M]y mother told me that. Then my aunt, then my grandmother, my father. These are people that cared for me--told me that I was okay and I would just have to get over it.”*

The family and friends’ associating depressive symptoms with biological or physical illness appeared to have complicated implications for how participants interpreted recommendations to stop suffering. One participant remembered that it seemed that friends’ and relatives’ failure to see depressive symptoms as on par with physical symptoms led to the advice, “*I knew that if I told them I was in [physical] pain, people would understand, but if I told them I was depressed, I'd be told just to ‘snap out of it.’”* Another participant recalled that a family member’s approach to stoically overcoming physical complaints seemed to lead to the recommendation that a similar approach should be used with depressive symptoms, “*I mean, my own father is, you know, military, 33 years hard ass. He would always say, ‘Snap out of it, you’ll be fine.’ ‘Come on, come on, men don’t…,’ oh, always. ‘Men don’t…,’ you know… ‘Hey, hey, you’ll be fine.’ Broken arms. Like, ‘Oh, you’ll be fine.’ I don’t know why all of a sudden (I was) telling people I don’t want to go anyplace and they’re going, ‘What’s wrong with you?’ And then family saying, ‘Snap out of it.’ You know, snap out of what? I don’t even know what I’m in.”*

### Feeling rejected

Participants’ memories of being labeled, judged, and lectured can be seen as implying some level of engagement by family and friends in participants’ experience of depression. Often, though, members of participants’ social network were reported to disengage, reject and shun the participant because he or she attempted to share his or her depressive symptoms. The reasons for friends’ and relatives’ reactions are likely varied and complex and our results reflect this variation. Some participants’ remembered only the rejection itself and not its cause. On such participant recalled, “*I, I generally have been abandoned when I express those feelings to family members and friends.”* Others appeared to have formulated assumptions about the reasons behind their family and friends’ apparent rejection. For example, one participant said, “*They’re afraid that it’s contagious. They’re afraid it’s contagious. They sit there like, ‘oh my god, I’m gonna get it too*.’” Another participant stated, “*They’re tired. They’ve been tired. They’ve been living with it. They’re tired. They’re fed up. They don’t have the strength anymore…when we go to them one more time and say, ‘Blah, blah, blah, blah, blah.’ You know, they don’t want to hear it. They, they don’t want to hear it.”* Yet another participant felt that family considered depression an inappropriate topic for family discussions and remembered, “*At one point in my life when I was really depressed, I tried to talk to my adult daughter who was a nurse, or my sister… and [they] say to me, you know, ‘I don’t want to hear it. That’s why you pay a psychiatrist. Go pay somebody to listen to you.’”*

Participants also remembered feeling rejected when attempting to discuss their depression treatment choices with family. Importantly, such rejection may have inhibited further depression themed discussion at the time and in the future. For example, one participant recalled, *“[M]y family didn’t agree with what I should do, which is to go seek counseling…. [S]o I just decided not to, you know, talk to them about my problems.”* Another participant similarly stated, *“I can’t open up and share…. When I started seeing a counselor, twenty something years ago, I did (open up and) … my sister was saying like, ‘Oh, we don’t believe in psychologists.’”*

## Discussion

Communication within families and other social networks around a stigmatized condition like depression is complex [28]. It is not surprising that the effects of persons’ interactions with their social networks can range from beneficial to detrimental [13-15,29,30]; and detrimental actions may result from motivations that range from well-intentioned to overtly malicious. Survey studies in chronic medical conditions have suggested that depressive symptom severity is correlated with negative social support [31-33]. Furthermore, depression-specific survey studies have investigated the perceived advantages and disadvantages of past attempts to seeking social support for depression [17] and the differences in perceived helpfulness of social support for a hypothetical future depressive episode between respondents with and without depressive symptoms [18]. The purpose of our analysis was to augment this nascent complementary literature, by providing a rich description of focus group participants’ recollections of negative social interactions and their reported impact on patients’ care-seeking and well-being. Our analyses provide nuanced descriptions, examples and a typology of less-than-helpful responses from family and friends that might undermine patients’ efforts to seek and follow through with treatment for depression in primary care.

There are two messages that arise from our results for primary care clinician teams to consider when providing care for depressed patients. First, it is evident from the poignancy of our participants’ recollections that these negative interactions with relatives and friends, especially for those viewed as *feeling labeled* and *feeling judged,* were accompanied by significant emotional pain. For some participants, this pain was still present years after those statements had been made. Such patients might be fearful that their interactions with others might recapitulate some aspects of the labeling and judgment they experienced from friends and family members. Thus, clinicians should take into account information from patients that might suggest prior expressed or potential harmful or hurtful statements from members of their social networks. By making initial inquiries about the potential for such a negative interaction, clinicians can know better when and how to advise both patients and other members of the care team about enlisting the support of patients’ families and friends in depression, when possible. We suggest beginning the conversation with a general question about how depression is viewed in the patients’ relatives and friends (e.g. *Has anyone in your family or social circle described feeling like you do now? How did they respond?*), and tailoring recommendations for invoking social support based on the presence or absence of actual or feared negative experiences such as *feeling labeled* or *feeling judged.*

The second message to arise from our analysis was participants’ descriptions of negative interactions with family and friends, particularly those viewed as *feeling lectured* and *feeling rejected*, leading to diminished communication about depression-themed topics. Both participants and members of their social networks inhibited such discussions, through avoidance, conflict or re-directing. The Theory of Planned Behavior [34,35] has been applied to depression help-seeking [36,37]. One of the theory’s most important contributions is its identification of the role of norms in motivating and shaping behavior. If the norms of patients’ social networks serve to inhibit disclosure [38], it is possible that the negative experiences categorized in our study and the fear of future similar experiences may lead to barriers in depression symptom disclosure to physicians as well, and/or affect adherence to treatment. While it may be overzealous to suggest that primary care clinicians can change the social norms to which patients are exposed, with awareness, clinicians’ words and deeds can enable patients to understand that the norms of their family or friends are not universal. With greater trust, patients may feel that they have at least one venue in which it is safe and permissible to discuss their depressive symptoms. By serving as one of many potential normative counter-weights, primary care clinicians can help patients interpret and respond to their often unforgiving social environments [16]. We suggest that clinicians begin the conversation in an open-ended way (e.g. *Have you discussed how you are feeling lately with family or others in your social circle? How did they respond*?), and following up with specific questions addressing patients’ fears of being *lectured* or *rejected* in the clinician-patient relationship.

### Strengths and limitations

The multi-centered nature of our data gathering methodology and the sample size that we were able to obtain are strengths of this study. Furthermore, participants’ comments arose spontaneously and unprompted in the context of a study designed to deepen the understanding of barriers to communicating with primary care practitioners about depression. It is possible that the interactions with family and friends reported by study participants were influenced by the depressive symptoms that participants were feeling at the time of the interaction or the later interpretation of those interactions influenced by depressive symptoms felt at the time of the focus group itself. The sample’s racial and ethnic diversity, while a strength, might also under-represent groups for which family has been reported to play a specifically important role in depression care [39,40]. We acknowledge that the experiences of our participants may differ from those of the general population of depressed primary care patients. In addition, focus groups may foster collective thinking which can lead to reinforcement of some themes and avoidance of others even when guiding questions are utilized. For example, friends and family members frequently play a helpful role in medical care [41,42] and the perspectives of the individuals implicated in participants’ recollections of discussions were not available. Also, the study was not designed to corroborate or provide causal links among implied motivations of members of the participants’ social networks, their reported actions and the effects of those actions on the study participants. Our recommendations for clinicians to engage patients in initial discussions of potential negative social support as part of a collaborative depression care approach must be viewed in the context of the spontaneous nature in which these recollections of negative social support arose, and our inability to expressly ascertain participants’ potential uptake of clinicians’ attempts to engage them about these negative experiences. The complementary nature of the multidisciplinary research team, made up of clinician-researchers (EFG, RLK, RE) and non-clinician mental health researchers (DP, CSC, PD), was integral to forming clinically relevant research questions and to tempering potential clinician-researcher bias in the data collection, analysis and interpretation. Furthermore, our recruitment strategy (self-selection into the potential participant study pool) and the discussions leading to informed consent minimized the potential for therapeutic misconception in participants of studies involving dual clinician-researchers. Lastly, data on validity of participants self-reported depression diagnoses were unavailable.

## Conclusions

While members of depressed patients’ family and other social networks often offer helpful support, our analyses have uncovered ways in which members of the social networks’ statements may undermine social support that might promote earlier and more effective treatment for depression. We have categorized the types of messages that these patients may hear in such a way that PCPs can identify such messages and explore in greater depth, either themselves or, ideally, via effective collaborative depression care [19,20] their patients’ potential social support context in delivering depression care [43-45]. Clinicians’ knowledge of patients’ sources of positive and negative social support can help enhance positive social influences and mitigate those that are unhelpful (with regard to disclosure of depressive symptoms and follow-through with treatment) or deleterious (with regard to further emotional trauma). Depending on the organization of primary care, time allotted to visits and other local and regional factors [46-48], these discussions might be conducted by the physician, a nurse-practitioner, a practice nurse, a social worker, a navigator or a case or care manager.

This qualitative report serves to open the door to several potential areas for further research. Depression care researchers should study in greater depth reasons why members of patients’ social networks present these negative messages to patients and in what circumstances patients actually modify their help-seeking behaviors in response to negative social support. Future research should also investigate the feasibility and effects of inquiry into family support in the context of primary care. Clinician training and implementation strategies for collaborative depression care in primary care settings [43,49] should also emphasize “First, do no harm” by alerting clinicians to the possibility that providers, no matter how well-intentioned, could deliver similar negative support messages as those delivered by patients’ family and friends [44,45,50].

## Competing interests

The authors declare that they have no competing interests.

## Authors’ contributions

EFG participated in the conception and design of the study; the acquisition, and interpretation of data; and led the writing of the manuscript. PD, DAP, and RME participated in the conception and design of the study; the acquisition, and interpretation of data; and critical revision of the manuscript. RLK obtained funding for the study; participated in the conception and design of the study; the acquisition, and interpretation of data; and critical revision of the manuscript. CSC participated in the acquisition and interpretation of data, and critical revision of the manuscript. All authors have read and approved the final manuscript.

## Authors’ information

EFG is Assistant Professor of Clinical Pediatrics and current Dean’s Scholar at the University of California, Davis, School of Medicine. PD is Professor of Psychiatry and Director of the Rochester Health Care Decision-Making Group. DAP is Associate Adjunct Professor of Medicine and Sociology and executive member of the Center for Healthcare Policy and Research at the University of California, Davis. CSC is Senior Community Health Program Representative in the Center for Healthcare Policy and Research at the University of California, Davis. RLK is Professor and Co-Vice Chair of Research in the Department of Internal Medicine at the University of California, Davis, School of Medicine. RME is Professor of Family Medicine, Psychiatry, Oncology, and Nursing and Director of the Center for Communication and Disparities Research at the University of Rochester Medical Center.

## Pre-publication history

The pre-publication history for this paper can be accessed here:

http://www.biomedcentral.com/1471-2296/13/64/prepub
